# AES Based White Box Cryptography in Digital Signature Verification

**DOI:** 10.3390/s22239444

**Published:** 2022-12-02

**Authors:** Piyush Kumar Shukla, Amer Aljaedi, Piyush Kumar Pareek, Adel R. Alharbi, Sajjad Shaukat Jamal

**Affiliations:** 1 Department of Computer Science & Engineering, University Institute of Technology (UIT), Rajiv Gandhi Proudyogiki Vishwavidyalaya (RGPV), Technological University of Madhya Pradesh, Bhopal 462033, Madhya Pradesh, India; 2 College of Computing and Information Technology, University of Tabuk, Tabuk 71491, Saudi Arabia; 3 Department of Computer Science and Engineering, Head of IPR Cell, Nitte Meenakshi Institute of Technology, Bengaluru 560064, Karnataka, India; 4 Department of Mathematics, College of Science, King Khalid University, Abha 61413, Saudi Arabia

**Keywords:** AES, white box cryptography, digital signature, attack

## Abstract

According to the standard paradigm, white box cryptographic primitives are used to block black box attacks and protect sensitive information. This is performed to safeguard the protected information and keys against black box assaults. An adversary in such a setting is aware of the method and can analyze many system inputs and outputs, but is blind to the specifics of how a critical instantiation primitive is implemented. This is the focus of white-box solutions, which are designed to withstand attacks that come from the execution environment. This is significant because an attacker may obtain unrestricted access to the program’s execution in this environment. The purpose of this article is to assess the efficiency of white-box implementations in terms of security. Our contribution is twofold: first, we explore the practical implementations of white-box approaches, and second, we analyze the theoretical foundations upon which these implementations are built. First, a research proposal is crafted that details white-box applications of DES and AES encryption algorithms. To begin, this preparation is necessary. The research effort planned for this project also includes cryptanalysis of these techniques. Once the general cryptanalysis results have been examined, the white-box design approaches will be covered. We have decided to launch an investigation into creating a theoretical model for white box, since no prior formal definitions have been offered, and suggested implementations have not been accompanied by any assurance of security. This is due to the fact that no formal definition of “white box” has ever been provided. In this way lies the explanation for why this is the situation. We define WBC to encompass the security requirements of WBC specified over a white box cryptography technology and a security concept by studying formal models of obfuscation and shown security. This definition is the product of extensive investigation. This state-of-the-art theoretical model provides a setting in which to investigate the security of white-box implementations, leading to a wide range of positive and negative conclusions. As a result, this paper includes the results of a Digital Signature Algorithm (DSA) study which may be put to use in the real world with signature verification. Possible future applications of White Box Cryptography (WBC) research findings are discussed in light of these purposes and areas of investigation.

## 1. Introduction

The employment of a piece of technology that is known as a digital signature is becoming more widespread as a result of its growing popularity. Its key duties are to identify and prevent any unauthorized changes to the data, as well as to verify the identity of the signature. The signing of legally binding contracts, the protection of software updates, and the use of digital certificates to ensure the security of online business transactions are some of the possible applications for digital signatures [1]. Digital signatures also have the potential to be used in a wide variety of other contexts as well. A public key white box is the single most essential example of a public key white box because of the fact that it uses public channels. This is in addition to the process of key establishment via insecure channels, which is also an important part of the equation. It is very necessary for the purpose of assuring the security of monetary transactions that are carried out across open or insecure networks. The bulk of the time, digital signature techniques are employed in white-box cryptographic protocols. This is to enable the provision of services such as entity authentication, authenticated key transfer, and key agreement [2].

In some contexts, the operation of digital signatures is comparable to that of handwritten signatures in their functionality. In particular, they provide a way to guarantee that a message that was sent to a particular user is genuine, in the sense that it really did come from the same person who said that they were the one who was responsible for sending the message [3]. This is due to the fact that they offer a means by which the identity of the individual who claimed to be the person responsible for delivering the message may be verified. Having said that, they provide a far higher level of functionality [4].

In a new computer environment, a large number of devices with limited capabilities are connected to the internet. The network enables the devices to communicate with one another, which results in an enhanced user experience. In today’s world, Internet of Things (IoT) technology makes it possible for numerous objects with limited resources and communication capabilities to communicate, compute, and make decisions inside a communication network. It is imperative that the security of limited end nodes be maintained in order to handle this new environment. Any and all private information is vulnerable to being compromised, accessed, and disclosed if adequate protection services are not in place. Because of the dramatic rise in system vulnerabilities, it is now much simpler to carry out attacks on information systems. This has the effect of making cyberattacks more likely. Because they do not have enough high-end security, an increasing number of companies are running into problems that put their work at risk; the root cause of these problems is a lack of resources. It is becoming more vital to safeguard information in order to guarantee its confidentiality, integrity, and availability as technology continues to improve and more people have access to more marketplaces. However, because of the limited resources of restricted devices, it is not a simple task to implement appropriate cryptographic functionality on such devices. “Lightweight Cryptography (LWC),” which is one of the state-of-the-art approaches, is able to offer security services as well as the necessary level of privacy for devices that make use of communication technologies. LWC refers to a kind of cryptographic technique or protocol that was designed specifically for use in situations with limited resources. When it comes to advanced communication technologies, lightweight cryptosystems are the best option for preventing various forms of malicious attacks and for enforcing the four primary security requirements of confidentiality, availability, integrity, and authentication. This is because lightweight cryptosystems are the least resource-intensive option. It is common knowledge that “Information Security” plays a significant part in the new era of information technology. This is necessary in order to adequately address lightweight cryptographic primitives, such as lightweight stream cyphers, hash functions, and low-resources devices for Internet of Things environments.

### A Description of The Digital Signature and How It May Be Used

A significant amount of time has passed during which individuals have been associating their identities with the documents that they sign using a variety of signatures. These documents include wills, contracts, and other legal documents. These signatures are seen on a wide variety of different sorts of papers. In order to ensure that a document was genuine during the Middle Ages, a nobleman would affix a wax seal on it. This would ensure that the document was authentic. It was generally believed that the noble was the only one who had the skills necessary to successfully recreate the emblem. However, this assumption proved to be wrong [5]. When used in current transactions, credit card slips need a signature to be written on the reverse of the slip. It is the responsibility of the salesperson to validate the signature by contrasting it with the signature that is already imprinted on the card. These strategies are now becoming irrelevant and inadequate as a result of the expansion of internet commerce and digital documentation. In today’s fast-paced technological world, the significance of information and communication systems is growing with the increasing significance and quantity of data that is transmitted in order to reduce operational costs and provide improved services. This is due to the fact that more data is being transmitted, which in turn means more data needs to be transmitted. This is as a result of the increased amount of data that is being delivered in order to accomplish these objectives. The expansion in the relevance of this phenomena has a direct association with the increase in the amount of data that is sent, and this correlation is a one-to-one relationship [6]. Unhappily, the vulnerability of systems and data is significantly expanding as a consequence of an increasing range of threats. These risks include the destruction, modification, and theft of systems and data, as well as unauthorized access and exploitation of data. All of the other data and information security processes are built on top of the white box concept, which acts as the basis for their creation. As a direct result of the fact that it makes the exchange of information easier and more expedient, the internet has been directly responsible for a fundamental shift in the manner in which business and transactions are conducted. This shift has been brought about as a direct result of the fact that it facilitates a quicker flow of information. Another example of a “white box” cryptographic method [7] is the usage of digital signatures.

**Figure 1:**
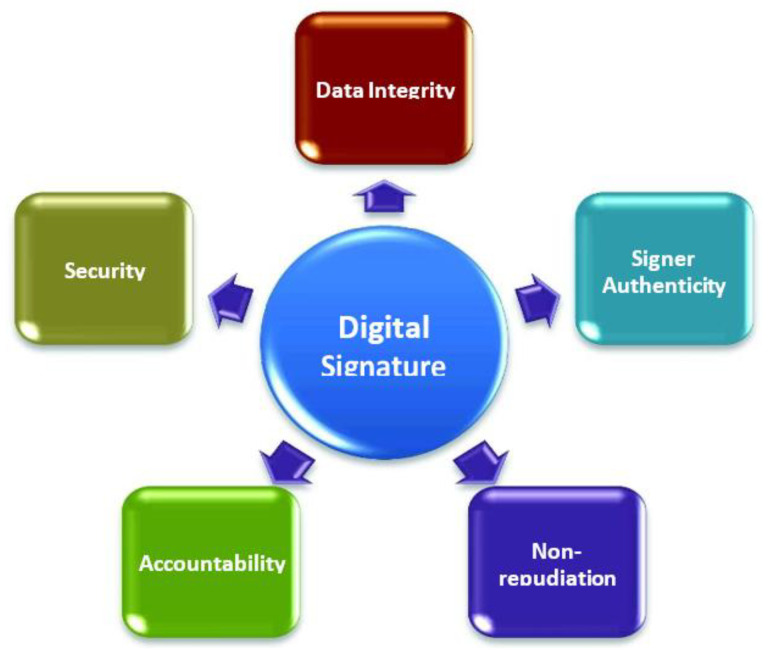
Security Advantages of Digital Signatures.

Using software that generates digital signatures helps organizations maintain the authenticity, accountability, data integrity, and non-repudiation of documents and transactions, as can be seen from the examples that have been presented. The use of digital signatures makes this doable and practicable (Figure 1).

Figure 1 represents the security advantages of the digital signatures. All of these goals were accomplished through the use of various encryption algorithms. One would be led to assume that, at this time, we are able to satisfy any and all security needs that may come up in real practice. This would be a mistaken assumption. In addition to encryption and the exchange of keys [8], there are a great many more security requirements that, from a technological standpoint, are regarded to be security services.

The digital signature will only be able to attribute the message to the signer if both the public key and the private key that are associated with the signer are both linked with the message [9]. In the event that the signer loses control of the private key, it will be impossible to duplicate the digital signature in a fraudulent manner.

Authentication of communications: in comparison to paper signatures, a digital signature can identify a signed message with a far better degree of accuracy and precision than traditional signatures. The comparison of hash results that takes place during verification reveals any efforts that were made to tamper with the message after it was signed, since it indicates whether or not the message has been changed [10].

This is a prerequisite for creating a digital signature [11]. In contrast, methods that use paper take a lot of time and require a lot of physical work. One example of this is the examination of signature cards.

Because electronic transactions drastically cut down on the amount of paperwork that is required, and also offer the possibility of significant increases in both efficiency and productivity, they are gaining significance in nations all over the world. This is one of the reasons why electronic transactions are becoming more important. As a direct result of this, digital signatures are on the cusp of becoming the primary method for establishing confidence across a wide variety of electronic transactions. This will likely cause them to enter the mainstream in the near future.

A checksum is what constitutes a digital signature, and the value of the checksum is determined by the amount of time that has passed since it was formed. It is computed by making use of a collection of criteria as well as a set of factors in order to make certain that the authenticity of the signature as well as the data can be verified.

The cloud has been effectively and promisingly utilized in all aspects due to its numerous characteristics and benefits, including low maintenance and computation cost. Customers are provided with a vast array of services by the CSPs, who operate gigantic datacenters. Customers are able to make use of the high-quality cloud services while also drastically cutting their massive investment expenditures by putting their local management systems on cloud servers.

The provision of online storage space in the cloud is the most important service that cloud providers provide. Customers are now able to move their files into the remote machine that is located on the cloud server as cloud storage continues to evolve. Customers are thereby liberated from the burden of thinking about the expenses of installing software and hardware in their own communities. The security risks associated with the cloud, which include data confidentiality, data integrity, data sharing, data robustness, and other related concerns, continue to increase in tandem with the development of the underlying technology.

Several different security models have been suggested in order to protect the data stored in the cloud and to protect the owners’ and users’ privacy.

However, there are still inefficiencies in the process of building a secure cloud storage system that includes all of the aforementioned requirements.

As a result, there is a need for a safe cloud storage system that maintains data confidentiality, data integrity, protects the data from unauthorized access, and supports data forwarding functionalities, all while maintaining the owners’ privacy.

Within the confines of this paradigm, it is taken for granted that both the communication endpoints and the computing environments can be relied upon. That is to say, it is presumed that the cypher execution (encryption/decryption, performed with a secret key) cannot be seen or altered in any way. Since an enemy may only access its functions, this model is often referred to as the Black-Box Model for this reason.

However, the assumptions that were made in the past may not be appropriate to the technology that is available now. Over the course of the last ten years, there has been a significant shift in the kinds of uses for which cryptographic methods are put into action. As a direct consequence of this, it is no longer safe to presume that the endpoints of the connection are trustworthy entities. This has a significant bearing on the implementations of cryptographic protocols’ levels of safety. 

There are a few different types of cryptographic models that may be found in published works, including the naked model, the plain model, and the (random) oracle model. The following are some samples of what a black-box model looks like.

If an implementation exists in a hostile environment, an opponent may be able to watch and tamper with the implementation to extract information about the cryptographic key. If the implementation is located in a hostile environment, it is possible that an adversary will be able to accomplish this. As a consequence of this, the techniques that were created in the past to evaluate the safety of cyphers may no longer be enough for the majority of the applications that are used today.

In the next two illustrations, we will demonstrate why the traditional black-box paradigm of encryption is flawed. 

Mobile agents are a kind of software that may be sent from a client computer to a distant server in order to be executed there. In many cases, they even have the ability to move freely inside a (public) network. Their objective is to complete the job that was assigned to them by their owner in a manner that does not involve any communication with the owner while they are working on the assignment. It has been suggested that they may be used as a way for carrying out transactions and retrieving information inside networks. Systems for purchasing airline tickets and those for conducting auctions online are two examples of typical mobile agent applications.

When it comes to the process of booking plane tickets, a mobile agent is sent by the company’s owner, who is looking for the least expensive trip from point A to point B. This agent will continue with the purchase of the ticket after searching the websites of a variety of travel agencies and airlines in an effort to choose the one that offers the best deal. The whole procedure has to be able to be carried out without requiring any participation from the owner, who ultimately wishes to obtain the electronic airline ticket.

It should come as no surprise that such a system is susceptible to a number of unusual dangers. To begin, there is a possibility that the servers used by travel agents and airlines may not operate in a trustworthy manner as end points. It is to their advantage to launch an assault on these mobile agents. For instance, to modify the code so that it reads “find my e 200 flight” rather than “find the cheapest trip,” or to compel the agent to purchase the ticket via their own servers. Second, in order to make the purchase of a ticket possible, the agent may need to be able to sign a contract or make a payment. Both of these things are necessary in order to complete the transaction.

Therefore, it is necessary to implement a public key cryptosystem so that a mobile agent may generate a digital signature for an electronic ticket. This will allow the mobile agent to use the ticket. However, since the mobile agent should not communicate with the owner during the process of purchase, the private signing key has to be held inside the code of the mobile agent. This is necessary. It is in the best interest of the malicious servers to obtain this private key information so that they may sign any electronic papers and, as a result, buy any items in the author’s name.

As a result, a logical issue emerges, which is whether or not it is possible to include secret data in software, despite the fact that the execution platform cannot be trusted. White-box cryptography is an approach that seeks to find an answer to this dilemma.

## 2. Study of White Box Cryptography

The concept that supports digital signatures is, at its most fundamental level, analogous to that which underpins handwritten signatures. Paper documents have been confirmed and authenticated using handwritten signatures for a very long time. This method is considered to be the most reliable. It is possible to employ a digital signature to offer assurance that the information was signed by the person who is claimed to have signed it. A digital signature is an electronic analogue of a written signature. A written signature may be converted into its electronic counterpart, known as a digital signature. In the realm of digital transactions, a handwritten signature is analogous to what is known as a digital signature. In order for a digital signature to be legitimate, it cannot be a fixed image; rather, it must be generated dynamically based on the information in the document that is being signed. This is one of the most significant distinctions between a handwritten signature and a digital signature. A digital signature may be altered at any time. It is used to verify the fact that a person pledged something that he or she cannot take back at a later time. Specifically, it is used to prove that a person would not break a promise made in the past. The promise cannot be revoked. When working with electronic documents, you need to employ a system that is capable of doing the same tasks as the electronic documents themselves. The practice of using digital signatures as a means of confirming and verifying the validity of electronic documents is rapidly becoming more widespread. When an algorithm for a digital signature is used, the output is a string of ones and zeroes that, when put together, constitute what is known as a digital signature [12]. The process of determining whether or not the information included in the document is accurate is referred to as “validation.” The process of verifying the identity of the person who sent a document is known as authentication. E-mail and other forms of electronic communication are the primary applications for the exchange of digital signatures. Software distribution and other applications that need to ensure the integrity of the data and authenticate the source of the data are examples of this. Other applications that require digital signatures include those that need to authenticate the source of the data. Digital signatures may also be used in other applications that require to verify the origin of the data, and this can be accomplished with the help of digital signatures. The wireless protocols, such as HiperLAN/2, each have their own set degrees of security, and the process of digital signatures is utilized in order to authenticate users.

Whenever data corruption or an unauthorized client is discovered during the process of data forwarding over dispersed servers, it is possible to simultaneously identify the servers that are performing improperly. This identification may take place at any time. This approach saves a lot of time and effort during the optimization of the number of cloud servers’ requests, which ultimately leads to congestion management between cloud servers. In spite of the fact that the scheme is quite effective and resistant to scheming failure, hostile data alteration assaults and even server colluding attacks are still possible in light of new discoveries and study. End-to-end security in the cloud storage environment is definitely a challenging job because malicious users can find a way to stop sending alert messages to the owner of the cloud storage organisation during the unauthorized access of the files. Because of this, end-to-end security is not always possible.

During a value-added online exchange of forward data over the cloud, the system is able to accomplish the integration of storage with secure forwarding and online alert notification to the service provider when unauthorized files are modified or accessed by malicious hackers. This is made possible by the combination of secure forwarding and online alert notifications. In addition, this approach is particularly useful for a number of other purposes, including (i) the management of congestion, and (ii) the optimization of the number of requests made between cloud servers.

Even though the results of the experiments show that the performance level of the proposed scheme is better in terms of throughput and storage server cost, there are still some problems that need to be solved. These problems include maintaining the data’s integrity, maintaining user anonymity, and determining how efficiently the users can retrieve the data.

It is also possible to use a digital signature to verify that the information that was signed has not been changed after it was signed. This may be conducted to ensure that the signed information has not been tampered with. A digital signature is a kind of electronic signature that may be used with any and all types of electronic transactions that can be conceived of [13]. A computer is able to produce what is known as a digital signature, which may then be saved in a digital format. The method of creating a digital signature, in addition to the outcomes that it offers, is significantly distinct from the techniques that are used to produce other kinds of electronic signatures. This is true both in terms of the process itself and the results that it delivers. Because of these qualities, digital signatures are superior to conventional signatures when it comes to their suitability for use in legal contexts.

In order to function properly, digital signatures are predicated on the application of certain mathematical procedures. Because these algorithms may be used as both a method for the production of signatures and a method for the verification of signatures, they can be utilized for any one of those purposes. The process of producing a digital signature on data is referred to as the generation process, whereas the process of determining whether or not a digital signature is authentic is referred to as the verification process. Both of these processes are referred to as the process of generating and verifying digital signatures. In order to sign and verify these objects, the person who has the signature needs to have access to a key pair, which consists of one private key and one public key. Only then will they be able to do so successfully.

**Figure 2:**
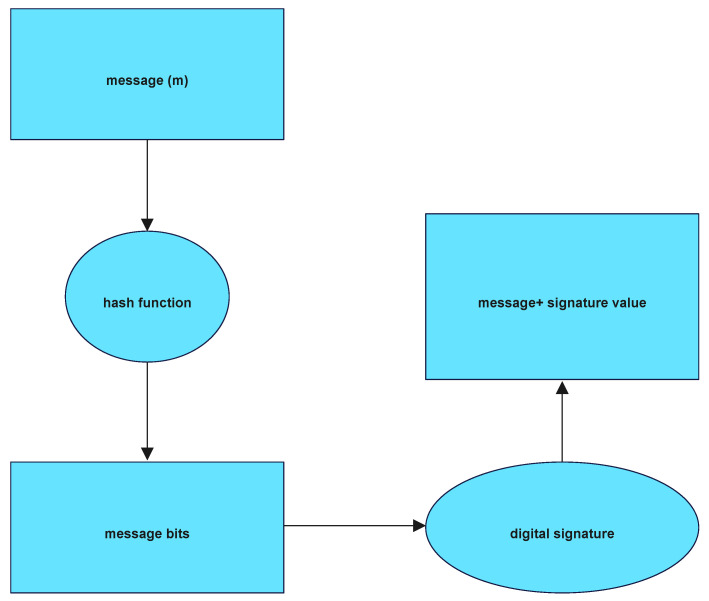
Signature Generation.

The fundamental premise behind digital signatures is that every signer has a private key that is completely one-of-a-kind to them. This is the principle that supports digital signatures. It is possible that the phrase contains an additional component of the key known as the public key. When a signer wishes to be sure that a document is authentic, his computer will create a bit string that is known as a signature for him to attach to the document. The person who has received this communication will next use his public key to verify the signature, as illustrated in Figure 2. Because of this, we can be certain that the bit string is accurate. If the receiver is satisfied that the document was signed by a person permitted to do so, then the receiver will accept the document. In the future, if the sender and the receiver are unable to come to an agreement about the validity of the document, a third party will review the signature, and, using the signer’s public key, they will verify the signature. This will happen only in the case that the sender and the recipient are unable to come to an agreement [14].

Figure 2 represents the signature generation. This was a novel idea that had not been discussed or considered before. This research is generally recognized as a landmark accomplishment in the field of white box, and with good reason.

It is possible to separate it into three separate operations, which are respectively referred to as the Generation of the Key, the Generation of the Signature, and the Verification of the Signature. The phase that is often referred to as “Key Generation” is the phase that is responsible for building the groundwork for it. Consequently, this phase bears the common name “Key Generation.”.

The primary objective of cloud computing is to provide a high number of calculations per second by holding vast amounts of data. These computations are based on traditional supercomputing or other forms of high-performance computing power. Therefore, in order to put this into action, networks from many servers are merged in order to put the client’s technologies into action at the lowest possible cost. The usage of virtualization techniques is widespread in the quest to improve the efficiency of cloud computing. It provides immense computational power and storage capacity, allowing its users or customers to experience the benefits of such services and capabilities without having to worry about the burden of any hardware or software installation and administration.

Cloud computing has introduced a number of new security concerns.

If the data activities and the transactions involving such data are not protected, it poses a significant risk of security breaches and other problems [2].

The provision of authentication, authorization, and access control methods is critical for the safety of data in the cloud environment [3]. This is due to the fact that the cloud opens the way for many businesses and organizations to utilize their services.

The management of the cloud [4] requires the use of a wide variety of software and procedures that are designed for the execution and monitoring of data and the processes linked with it. In addition to this, it gives users the assurance that the cloud resources will operate well and that they will have an easy time working with customers.

The use of cloud computing is becoming more popular among businesses of all sizes because it makes it possible to safely and easily exchange data and resources over the internet while also ensuring their privacy.

## 3. Key-Pair Generation

When dealing with cloud storage, one of the most difficult challenges is building a data-sharing system that is both private and secure for the ever-changing membership of a group. After this, a suggestion was made for a method of securely exchanging data that would not call for the use of any communication channels in order to disseminate the necessary keys. The revoked members are unable to access the data files stored in the cloud. This method utilizes a polynomial function in order to achieve both a fine-grained access control and a secure user revocation, which enables the data files to be protected from unauthorized access. The data that is shared throughout the group is accessible to each and every member of the group. Additionally, the system is able to support dynamic groups, which means that the private keys of the users do not have to be recalculated or changed whenever a new member is added to the group or an existing user is expelled from the group. This is because the system is capable of supporting dynamic groups. For the aim of transmitting sensitive information inside the cloud, it has also been recommended to use an ElGamal cryptosystem that is coupled with bilinear pairing. They used a method of encryption that is known as incremental 9, which entails separating the data into blocks and then encrypting each of those blocks one at a time. This was performed in order to protect the confidentiality of the information. The generation of the key, as well as the operations of re-encryption and decryption, were all handled by a reputable third party. This group is also responsible for handling all of the jobs that need a lot of computing power. It was also proposed that a method for the safe sharing of data inside a group that is based on the idea of shared key derivation may be used. However, due to bilinear pairing and collusion assaults, there is a difficulty with the computational cost of all of these alternative techniques. As a result, it is of the highest importance to work toward the development of a secure means of data sharing inside the cloud that does away with the various problems involved. When it comes to meeting the criteria for searching efficiency and keeping the anonymity of the content being retrieved, as well as its owner and the person doing the retrieving, the searchable encryption approaches that are now in use have a few flaws that need to be worked out. In addition, a cryptographic function is required in order to successfully recover the encrypted data content as well as validate the keywords that were used to encrypt the data. When it comes to the exchange of data, the development of encryption keys is what ultimately defines how successful the different data protection solutions are. When a group of files has to be made accessible to several users, each user will need their very own individual encryption and search key in order to be able to access the files. It will be necessary to write down and save these user-specific encryption and search keys in order to utilize them at a later time. However, in order for users to perform a search, they need to provide the same number of keyword trapdoors that serve as keys to the database. The completion of this step is required before utilizing the communal cloud storage. This underscores the necessity for a secure storage and communication system, which makes it difficult to carry out the plan as it was originally conceived. As a direct consequence of this, a KASE [10] was proposed. This KASE makes it feasible for the owner to disclose the key to the user, but in order to access the shared data, the user is required to submit a single trapdoor. The process seems to be risk-free and maintains the data’s anonymity while it is carried out. Although cloud storage has the potential to be a helpful function that the cloud provides, it also poses a number of additional problems about the security of one’s data. When utilizing cloud computing to exchange data, such data may be vulnerable to a range of attacks and threats coming from both inside the business and from outside the organisation. These attacks and threats might come from either party. As a result, developing a secure cloud storage system that enables tasks such as data transmission, data integrity, data privacy, and access control may be an extremely challenging endeavor. As a direct consequence of this, a method known as Secure Data Exchanging in Cloud (Seda SC) was created with the intention of facilitating the safe transfer of data inside the cloud. Each user has access to two separate keys, one of which is independently shared by the user, and the other of which is shared by the cryptography server. Both of these keys are used by the user. By using symmetric encryption, the approach safeguards any sensitive information that may be present. Additionally, it enables data forwarding without the use of the bilinear Diffie–Hellman problem (BDH) or the elliptic curve method. Additionally, it eliminates the risk of attacks and threats posed by insiders through the implementation of forward and backward access control.

In order to generate key pairs that may later be used in digital signatures, a piece of technology known as RSA is used in the process. The strategy known as the public key white box method is the one that is used the majority of the time in each and every area of the planet. The fundamental concept that underpins this is that multiplying prime numbers is not too difficult, but that factoring prime numbers is a task that requires a great deal more effort and thought. The computation of multiplication can be performed in a time that is polynomial, but the time required to factor may expand at a rate that is exponentially proportional to the size of the numbers being factored. Multiplication can be performed in a time that is polynomial. The operation of the algorithm is described in detail in the following paragraphs [15]:

Figure 2 presents the data flow diagram for the generation of key pairs, which may be found further down the page.

### 3.1. Sign Generation

With the message m in hand, we use the appropriate hash function H (MD5, SHA1, or SHA2) to generate the hash result M = H (m).

When we want to sign a message, we use M n to calculate a signature that looks like this: Signature d S = M (mod n), where d is the signer’s private key.

Figure 2 shows the data flow diagram of Signature generation module in application.

#### 3.1.1. Sign Verification

1. We use the digital signature S to validate the message m by computing e M = S (mod n), where he is the public key of the signer.
2. After that, we obtain M’ = H(M), which we then contrast with M.
3. If both of these things are the same, then the message is genuine; otherwise, it has been tampered with.

**Figure 3:**
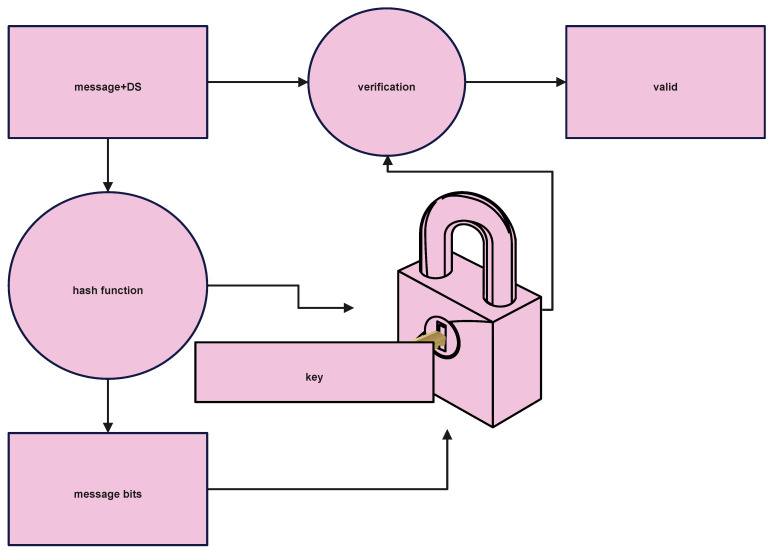
DFD of Digital Signature Verification Module shows the data flow diagram of Signature verification module in application RSAAPP.

Figure 3 represents the digital signature verification module of the proposed work.

#### 3.1.2. Sign Verification

The Signatures are able to be created as well as validated with the assistance of the Digital Signature Authority (DSA).

(1) $$p_{n}\left(i\right)=\frac{n_{i}}{n},0\leq i<L$$

$p_{n}\left(i\right)$ represents the message function in the input data. L represents the length of the message in the information system. To generate a digital signature, you will need a private key, and you will continue to make use of this key throughout the process of creating the signature. Signature A public key is what is used throughout the verification process [16]. This public key is connected to the private key in some way, but they are not the same thing at all. Every user makes use of their own unique set of private and public keys. It is assumed that public keys were used to sign data that was preserved and data that was transmitted. This applies to both types of signatures. Any independent party may verify a user’s signature if they have access to the user’s public key and utilize it. The only person who is able to produce a signature for a user is someone who has possession of the user’s private key [17].

Both the signer and the data that has been signed are subjected to the Data Signing Authentication (DSA) protocol’s stringent checks to ensure that they are genuine. It is also feasible to use the DSA to show to a third party that the data in question was in fact signed by the person who created the signature. This may be performed by proving to the third party that the data in question was signed by the person who generated the signature.

(2) $$DSA{\left(i\right)}_{s}=\overset{i}{\underset{j=0}{\sum }}ixp_{x}\left(j\right)$$

*x* is the created signature in the information. It is difficult to achieve interoperability since different signature systems are not compatible with one another. This problem has already started to be handled in a variety of different ways, which is good to see. In order to take full advantage of the benefits brought about by incremental changes to anything, the process of making such improvements must be ongoing. The padding format is the most important area for interoperability; nevertheless, in order to achieve full interoperability, additional aspects must also be compatible. The key area for interoperability is the padding format itself. These additional considerations include the selection of a hash function to use as well as the variety of key sizes that are available.

(3) $$p{\left(i\right)}_{y}=iK$$

Although p represents the attack in RSA, which provides a high degree of security, one of the known techniques of attack is termed “brute force,” and it is possible that this approach may be used. The easiest way to protect yourself from this attack ($E_{attack})$ is to make the key bigger so it is harder to type in. Nevertheless, there is a chance that this tactic may lead to a number of problems, such as the following: Increased processing time—approximately, decryption time increases 8-fold when key sizes double; computational overheads—the computation required to execute the public key and private key conversions; and an increased probability that the key may be compromised.

(4) $$E_{attack}=\int_{0}^{1}x\left[E_{int}\left(p\left(s,t\right)\right)+E_{eut\;}\left(p\left(s,t\right)\right)\right]ds$$

The fact that RSA key storage, for both private and public keys, takes a significant amount of memory for archiving reasons is one of the primary factors (Y(k)) that has led to a growth in the demand for key storage. As a direct consequence of this, there is an immediate and compelling need to place a greater focus on the formation of key pairs and the storage of private keys. It is sometimes necessary for industry, banking, and government standards to be different; nevertheless, the padding format itself does not always need to be a source of incompatibility.

(5) $$Y\left(k\right)=\frac{1}{N_{A}}\overset{N_{A}}{\underset{n=1}{\sum }}\;E_{attack}{\left|h_{k}\left(n\right)\right|}^{2}=\tilde{p}\left(k\right)+{\tilde{\sigma }}_{I}^{2}\left(k\right)$$

### 3.2. Hash Functions

The so-called “hash function” is a crucial component of a variety of white box cryptography systems.Only within the world of mathematics is it even possible to think of the hash functions ($h_{k}\left(n\right)$) that are utilized in white box. Because hash functions do not need a key, they are often used in the construction of protocols and are considered an essential part of white box cryptography.

(6) $${\hat{\sigma }}_{I}^{2}\left(k\right)=\frac{1}{N_{A}}\overset{N_{A}}{\underset{n=1}{\sum }}x{\left|h_{k}\left(n\right)-\sqrt{p\left(k\right)}\alpha_{1k}\left(n\right)\right|}^{2}$$

All of these titles refer to the same thing. The digest of a particular message may be compared to the fingerprint of that message; that is, the digest is a one-of-a-kind representation of the message. You can think of the digest as the fingerprint of the message. It is feasible to use a hash function to make various algorithms more efficient provided that the function satisfies certain constraints about its non-invertibility. These characteristics are as follows: A message may be of any length, but a hash will always have the same length, since the typical functioning of a hash function is to produce a hash with the same length every time. Given the hash, it is impossible to find a message that has that hash; in fact, it is impossible to discover any information that can be utilized about a message that has that hash, not even a single byte of it. Data cannot be used to discover a message that has that hash. After a message has been digested or compressed using hash functions, which results in the message having a consistent length, the message must be signed in a manner that makes it extremely difficult to locate additional messages with the same hash. This is performed so that the integrity of the message can be maintained (so the signature would not apply easily to other messages). Because it creates a message digest, which is a compact and one-of-a-kind representation (akin to a more sophisticated checksum) of the whole message, hashing is a fundamental approach that is employed in digital signatures. This is due to the fact that it generates a message digest. Since hashing is a one-way encryption method, it is not possible to reconstruct the original message from the hashed version using hashing methods. This is because hashing is a one-way encryption method. The digest, which is often quite a bit smaller than the message itself, will be the target of the application of the digital signature. The following is a list of the primary justifications for constructing a message digest: (1) The authenticity of the message that is being sent is maintained, and any attempt to change the content of the message will be promptly uncovered. (2) The message itself will take up a far larger amount of space than the digest would. (3) Hash methods are far quicker than any other kind of encryption scheme (asymmetric or symmetric).

(7) $$D^{2}\left(x,y\right)=\overset{m}{\underset{x=0}{\sum }}\;\overset{n}{\underset{y^{\prime }=0}{\sum }}\;{\left[f\left(x^{\prime },y^{\prime }\right)-t\left(x^{\prime }-x,y^{\prime }-y\right)\right]}^{2}$$

t(x,y): template, M,N size of the template

(8) $$\overset{M}{\underset{x^{\prime }=1}{\sum }}\;\overset{N}{\underset{y^{\prime }=1}{\sum }}\;{\left[f\left(x^{\prime },y^{\prime }\right)-t\left(x^{\prime }-x,y^{\prime }-y\right)\right]}^{2}=\;\sum_{x^{\prime }=1}^{M}\sum_{y^{\prime }=1}^{N}f{\left(x^{\prime },y^{\prime }\right)}^{2}+\;\sum_{x=1}^{M}\sum_{y=1}^{N}t{\left(x^{\prime }-x,y^{\prime }-y\right)}^{2}-\;2\sum_{x^{\prime }=1}^{M}\sum_{y^{\prime }=1}^{N}f\left(x^{\prime },y^{\prime }\right)t\left(x^{\prime }-x^{\prime }y^{\prime }\;-y\right)\;\;\;Correlation:\;\;\;\;convolution\;of\;f\left(x,y\right)\;\;\;with\;t\left(-x,-y\right)$$

A hash function is a function with the form: D R, where the domain D is equal to 0, 1*, which indicates that the elements of the domain are binary strings of variable length; the range R is equal to 0, 1n for some n 1, which indicates that the elements of the range are binary strings of fixed-length; and the domain D is equal to 0, 1n for some n 1, which indicates that the hash function is a domain-range function. A hash function is a function that takes as an input a message M of an arbitrary size and produces a fixed-length hash result h of size n, where n is the size of the output in its entirety. When the space covered by a hash function’s domain is limited, that particular function is known as a compression function. In other terms, a compression function is a hash function that accepts a message of a set length as its input and generates a message of a shorter fixed length as its output. This kind of hash function is also known as a “compression function”.

In cryptography, a hash function is referred to as a white box cryptographic hash function H if it has the additional security qualities listed below:

1. H should be able to handle data blocks of any size when it receives them as input.
2. H should consistently provide an output of the same length, irrespective of the length of the data it gets as input.
3. The behavior of H should be consistent with a random function while yet being predictable and easy to reproduce. It is anticipated that H will accept inputs of any length and generate random strings of lengths that have been specified in advance as outputs. Determinism should be applied to H, and it should be repeatable in an efficient manner. This means that H should always produce the same output when given the same input, and that this input should never change.
4. The name given to this phenomenon is the one-way resistance, however it is also often called the pre-image resistance. It only indicates that the message’s hash value should not be utilized to reconstruct the message from its initial state. This is the only thing that it says.
5. If you are provided with a message M1, it is mathematically impossible to locate a message M2 that is shorter than M1 and has the same hash value as M1 at the same time (M2). This characteristic is also known as the second preimage resistance property or the weak collision resistance. Both of these names are used sometimes.
6. It is mathematically impossible to find any two distinct messages (M1 and M2) that, when combined, provide the result H(M1) = H for any given set of input parameters. [Cognitive impossibility] (M2). This characteristic is often known as the strong collision resistance.

These properties are required in order to prevent or resist certain types of attacks that may render an otherwise effective and secure white box cryptographic hash function useless. Dictionary attacks, buffer overflow attacks, and message authentication code assaults are a few examples of the types of attacks that fall under this category. A white box cryptographic hash function should not only provide a “digital fingerprint” of a message M that is unique and should also provide excellent collision resistance, but it should also be very sensitive to even the tiniest change in the input message. Another requirement for this type of function include that it should provide excellent collision resistance. Therefore, even a little change to the input message, such as adding or removing a single digit, should result in a considerable shift in the hash value of the message. This is because hash values are calculated using a one-way function. It is essential to keep in mind that a message in this context may arrive in the form of a binary text file, an audio file, or a programmed executable.

It is not required to keep the hash function a secret in order to maintain its safety; rather, the safety of the hash function is derived from its ability to create collision-free one-way hash values as well as from the fact that it is not sensitive to collisions. The occurrence of a collision indicates that two or more separate messages have produced the same hash value. It is possible to utilize hash functions in conjunction with a key, which may be either symmetric (shared key) or asymmetric. When this is done, the function is referred to as a message authentication code, or MAC. Hash functions that are employed without a key have been the primary topic of discussion up to this point; however, hash functions may also be used in conjunction with a key.

#### 3.2.1. Standard White Box Cryptographic Hash Functions

White box cryptographic hash functions may take a wide variety of shapes and be configured in a wide variety of ways. Hash functions may be broken down into two major categories, and users can choose any one. Message Authentication Codes, more often abbreviated to MACs, are another name for hash functions that must first be provided with a key before they can carry out their calculations. Hash functions whose computation does not depend on a key are more often known as un-keyed hash functions, or just hash functions, along with hash functions whose calculation does not rely on a key.

Every single one of the well-known hash functions base their operations on either a block cypher or modular arithmetic. However, before we go into the mechanics of them, let us take a look at a well-known example. The Merkle–Damgård construction is now the most frequent and commonly used method for creating hash functions. Let us have a look at it.

**Figure 4:**
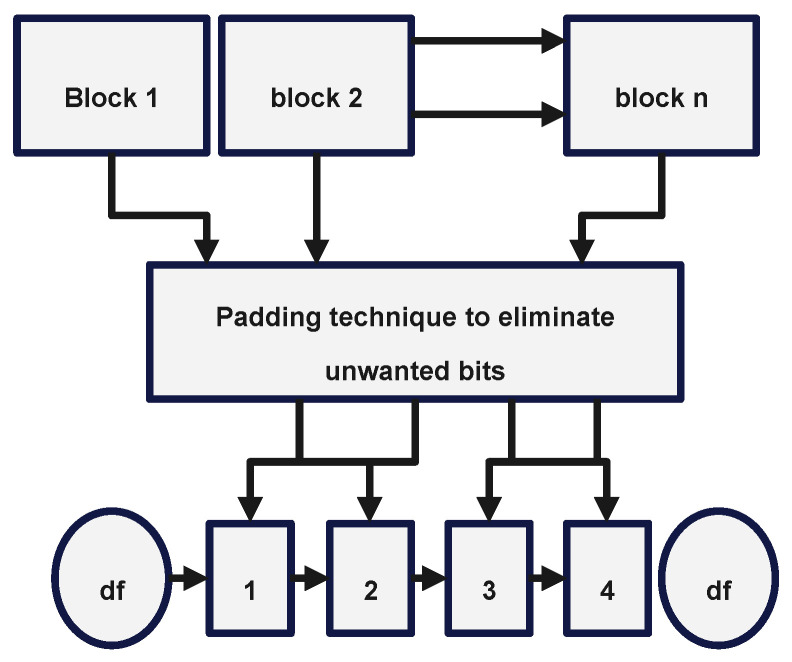
The Merkle–Damgård construction.

Its purpose is to produce a value of a predetermined length, regardless of the length of the value it receives as input. The Merkle–Damgård structure received its name from the two people who came up with the idea for it. Figure 4, which may be seen further below, illustrates the process that should be followed.

As represented in the Figure 4, the Merkle–Damgård structure may be broken down into its primary components, which are listed in the following paragraphs:

Initialization Vector, which is also known by its other name, Initial Value, is a value that cannot be changed in any manner and serves as the chaining variable for the very first iteration of the process. Vector is also known by its other name, Vector, which means Initial Value.

The compression function, or the one-way hash function, is either purpose-built specifically for the act of hashing, or is based on a block cypher. The hash function for general use is denoted by the letter g. The compression function, in the vast majority of cases, calls for an input of a length that has been determined in advance, and it ultimately generates an output that has the same length as the input. When a function known as “finalization” is applied to the output, we say that the output has been “finalized.” This function often makes the output value that was created by the most recent iteration utilize fewer bytes of storage space than it did before.

A hash of the message, which is also referred to as the message digest in certain instances. In order to begin the process of hashing a message, the whole message that has to be hashed must first be segmented into n length-based blocks that are identical to one another before the process can start. The limitations that are specified by the compression function f will determine the actual length that the message blocks need to be in order to work properly. After that point, the length of the message will continue to be padded in such a manner that it will always be a multiple of another number or a certain quantity. This process will continue until the message reaches the desired length. Because of this, it is easier to ensure that the padding length bits are always inserted accurately. The algorithm that does the compression works its way through each and every block, subjecting it to the exact same processing, which is performed in a repeating manner.

A message block and a chaining variable are required for each step or iteration of the compression function. These are referred to as the inputs. Before the function may continue, certain inputs absolutely need to be given. These are always essential components to have available. The Initialization Vector, which is also known as the IV, will perform the duties of the chaining variable during the very first iteration of the process. This will be the case even if the IV has another name. It is supplied as an input to the compression function, together with the original message block that is being compressed as a part of the process of compression. In the second iteration of the procedure for chaining, the output of the compression function f is used as the chaining variable. This is performed in order to maximize efficiency. In the process of chaining, this takes place at the very first iteration. When we are on the *i*th iteration, the result of the compression function f is used as the chaining variable for the *i*th plus one iteration. This continues until we have reached the last iteration, at which point we stop the iteration process.

The computation of the message digest may be described in a broad sense as follows for a message M that is made up of t blocks M0, M1, and Mt−1:

(9) H0=IV,  

(10) Hi+1=f(Hi, Mi) for 0 ≤ i<t   

(11) H(M)=Finalisation(Ht)

The Merkle–Damgård architecture is, in point of fact, the single most fundamental reason why thinking of hash functions as black boxes is incorrect, and it is the reason why doing so is wrong. It is illogical to expect additional security guarantees on top of that, since the iterative design was built to fulfil a particular and rather simple purpose, which is to expand the domain of a collision-resistant function.

The MD5 method is used for the purpose of determining whether or not data has been tampered with. It accomplishes this goal by producing a message digest consisting of 128 bits from the data that is entered, which may be any length of message. It is stated that this particular message digest is as distinctive to the data as a person’s fingerprint is to them. These programs are based on a public key cryptosystem and demand that huge files be compressed using a manner that is safe before they will encrypt the data using a secret key. One of the algorithms that is capable of meeting this condition is called MD5. In addition, the standard specifies that using a message digest to generate a Rivest-developed MD5, which is the third iteration of this message digest algorithm, is “computationally infeasible.” This is because MD5 is the result of the third iteration of Rivest’s message digest method. MD2 was developed for 8-bit devices, but MD4 and succeeding formulae are optimized for 32-bit processors. The architecture of all three are quite similar, however MD2 was designed to work best with 8-bit computers, whereas the formulas that follow were developed to work best with 32-bit environments. The MD5 technique is an enlargement of the MD4 algorithm, which, after being submitted to an in-depth analysis, was found to be efficient but maybe not totally secure. The MD5 method was developed as a result of this discovery. The MD5 approach, on the other hand, is not even close to being as fast as the MD4 algorithm, but it offers a far better degree of assurance about the data’s integrity. This is a classic example of the trade-off that takes place whenever safety is prioritized above velocity.

**Figure 5:**
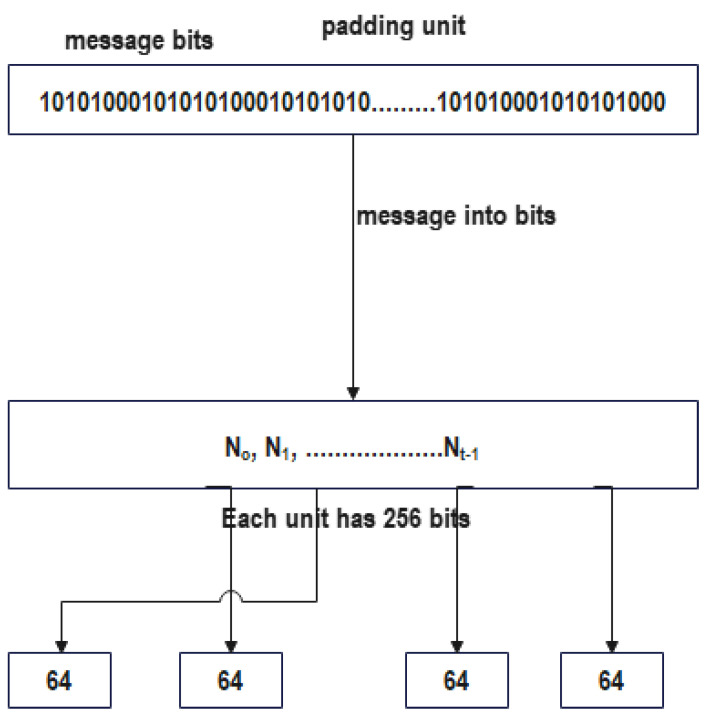
The MD5 Algorithm.

Figure 5 represents the MD5 Algorithm of the proposed work. Let us begin by making the premise that we have a b-bit message that we can use as input, and that we want to acquire the message digest for the data that we have. b is an arbitrary positive integer in this context; it may have the value zero, it does not need to be a multiple of eight, and it can be whatever size that you want it to be. The pieces of the message that we think we can make out written down look somewhat like this:

m_0 m_1… m_{b−1}(12)

It is necessary to carry out the five steps that are listed below in order to successfully compute the message digest of the message.

**Step 1.** Append Padding Bits

When the modulo 512 operation is performed on the message, its length (in bits) has been “padded” (stretched) in such a way that it corresponds to the value 448. In other words, the length of the message is raised until it is so long that it is just 64 bits shy of being a multiple of 512 bits. The padding process consists of the following stages being taken: The message is padded by first adding a single bit with the value “1,” and then by adding bits with the value “0,” in such a way that the length of the padded message, measured in bits, becomes equal to 448, modulo 512. Attached data may range from a single bit up to 512 bits in length. The minimal amount of data is one bit.

**Step 2.** Append Length

The outcome of the step before this one includes a representation of the value b that uses all 64 bits of the computer’s memory. This number indicates how long the message was before the padding bits were added to it. In the very unlikely event that b has a value greater than 264, the value of b will be lowered such that only the lowest 64 bits of its representation are used. (These bits are connected as two 32-bit words, and, in accordance with the standards that were in place before, they are inserted low-order word first.).

After this point, the length of the final message (after padding it with bits and with b) is an exact multiple of 512 bits in length. The fact that the length of this message is equal to an exact multiple of 16 (32-bit) words demonstrates the importance of the information it contains. The words that make up the final message will be represented by the notation M [0 … N−1], where N is a number that is a multiple of 16, and M is the message itself.

**Step 3.** Initialize MD Buffer

A buffer that is composed of four words is used in the computation of the message digest (A, B, C, and D). In this particular scenario, all four of the registers, A, B, C, and D, are each of the 32-bit kind. To initialize these registers, you will need to use the values that are shown below. These values should be written in hexadecimal with the low-order bytes appearing first.

The next step is to break the message down into 16-word chunks and process it.

To begin, we are going to build four auxiliary functions. Each of these functions will have the capacity to take in three 32-bit words as an input and will produce one 32-bit word as an output.

F(X, Y, Z) = (X AND Y) OR ((NOT X) AND Z)

G(X, Y, Z) = (X AND Z) OR (Y AND (NOT Z))

H(X, Y, Z) = X XOR Y XOR Z

I(X, Y, Z) = Y XOR (X OR (NOT Z))

The letter F is used as a conditional statement at each bit position, and the statement that it represents reads as follows: if X then Y else Z. Since XY and not(X)Z would never have 1 s in the same bit region, the function F may have been built by using the plus sign instead of the or sign. This is due to the fact that 1 s would never be present in both of these locations. It is worth mentioning that if the bits of X, Y, and Z are independent and unbiased, then each bit of F(X,Y,Z) will likewise be independent and unbiased. This is a truth that you should keep in mind since it is interesting.

The functions G, H, and I are similar to the function F in the sense that they produce their output by functioning in “bitwise parallel” to generate their output from the bits of X, Y, and Z. If the bits of X, Y, and Z that correspond to each other are independent and unbiased, then each bit of the functions G(X,Y,Z), H(X,Y,Z), and I(X,Y,Z) will likewise be independent and unbiased. To put it another way: the bits of X, Y, and Z that correspond to each other are independent and unbiased. It is essential to bear in mind that the actual meaning of the function H is the “xor” or “parity” function of its inputs.

In this step, the sine function is employed to build a table with 64 components that is referred to as T[1 … 64]. This table is used. Let us denote the item in the table that is the i-th one by using the notation T[i]. This item is equivalent to the integer component of the product that is 4,294,967,296 times abs(sin(i)), where I is measured in radians. In other words, this entry equals 4,294,967,296 times abs(sin(i)).

The following is a comparison between MD4 and MD5 with regard to the differences between the two:

1. From this point on, there will be a total of four rounds.
2. At this stage in the process, each step has its very own unique additive constant.
3. In order to reduce the degree to which the function g in round 2 is symmetrical, its previous notation—which read (XY v XZ v YZ)—has been changed to the current notation—which reads (XZ v Y not(Z)).
4. The result of the step that came before it is now incorporated into each step that comes after it. This results in a quicker onset of the so-called “avalanche effect.”
5. In rounds 2 and 3, the order in which input words are made available is shuffled around in an effort to develop patterns that are less similar to one another than the patterns that were formed by the same words.
6. The amounts of shift that are used in each round have been approximately tweaked in order to produce a more immediate “avalanche impact.” The shifts that take place in the game from round to round are very identifiable.

##### Security of MD5

This property gives the MD5 algorithm its name. This is one of the most helpful qualities that the algorithm has. This is because of the way that hashing works. According to Ron Rivest’s theory, the level of security provided by MD5 is the highest possible for an algorithm that uses 128 bits to hash data. According to Rivest, there must be a total of 264 procedures carried out before it is possible to discover two messages that have the same hash value. This is due to the birthday paradox, which was covered in the preceding section; identifying a message based on its related message digest will need 2128 operations. The pre-image attack is the name given to this particular kind of assault.

In order to perform the duty of calculating a message digest for a message or data file that is submitted to the SHA-1 algorithm in the form of input, the SHA-1 algorithm is utilized. It is helpful to think of the sending of the message or the storing of the data as a string of bits. The length of the message may be directly correlated to the number of bits that it contains (the empty message has length 0). If the total number of bits in a message is a multiple of eight, then we may consider using the hexadecimal version of the message in order to conserve space. The purpose of message padding is to ensure that the total length of padded messages is always a multiple of 512 characters. This may be accomplished by ensuring that the total number of characters in each message is 512. During the process of generating the message digest, SHA-1 moves methodically through the 512-bit blocks in the specified sequence. In a nutshell, in order to create a padded message with the length 512 * n, it is essential to attach a “1” followed by m “0”s followed by a 64-bit integer to the very end of the message. This is performed in order to build a padded message with the length 512 * n. The length of the first message is indicated by the 64-bit integer l, which represents the length. Following that, the SHA-1 algorithm will act on the padded message by breaking it up into n separate 512-bit blocks.

If n is more than zero, the message will be prolonged by padding it with 16 times n words. The padded message is represented as a string of n blocks, each of which contains 16 words, with block M1 having the first letters (or bits) of the message. The message is padded with zeros and spaces. The aggregate name for these blocks is M1 through Mn, and they are listed in this order.

The processing functions need to be prepared before moving on to the second stage. The SHA-1 algorithm makes use of a sequence of logical functions that are designated by the names f0, f1,…, f79.

The third step involves preparing the processing constants. The SHA-1 algorithm uses a string of constant words that runs from K(0) to K(1) to… K. This string of constant words proceeds in order (79). The following is how these values can be expressed using hexadecimal notation: K = 5A827999 (0 = t = 19) Kt = 6ED9EBA1 (20 <= t <= 39) Kt = 8F1BBCDC (40 <= t <= 59) The expression CA62C1D6 is equivalent to the value Kt (60 = t = 79).

The message digest must then be computed, which is the fourth stage. During the process of computing the message digest, the padded message is used in its completed form. In addition to a sequence that contains eighty 32-bit words, the method makes use of two buffers, each of which is formed of five 32-bit words. Each buffer is used in conjunction with the sequence. The words that are included inside the first buffer of five words have been assigned the letters A, B, C, D, and E as their labels. The following are the labels for the words that are included in the second five-word buffer: H0, H1, H2, H3, and H4. The 80 words that make up the sequence have been assigned designations beginning with W0 and continuing through W79. There is also a spot utilized as a temporary storage place for a single word.

In order to generate the message digest, it is necessary to process the 16-word blocks M1, M2,…, Mn that were defined in step 1. These blocks must be processed in the order that they were listed in step 1. There are a total of eighty distinct phases involved in the processing of each Mi. The “Hi” are initialized in the following way before to commencing the processing of any blocks: H0 equals 67452301, H1 equals EFCDAB89, H2 equals 98BADCFE, H3 equals 10325476 in hex, and H4 equals C3D2E1F0.

The projects M1, M2,…, and Mn are currently being worked on. The following is the method that will be used by our company for processing Mi: a. To begin, take the word Mi and divide it into 16 words using W0, W1,…, W15, with W0 functioning as the leftmost word.

**Figure 6:**
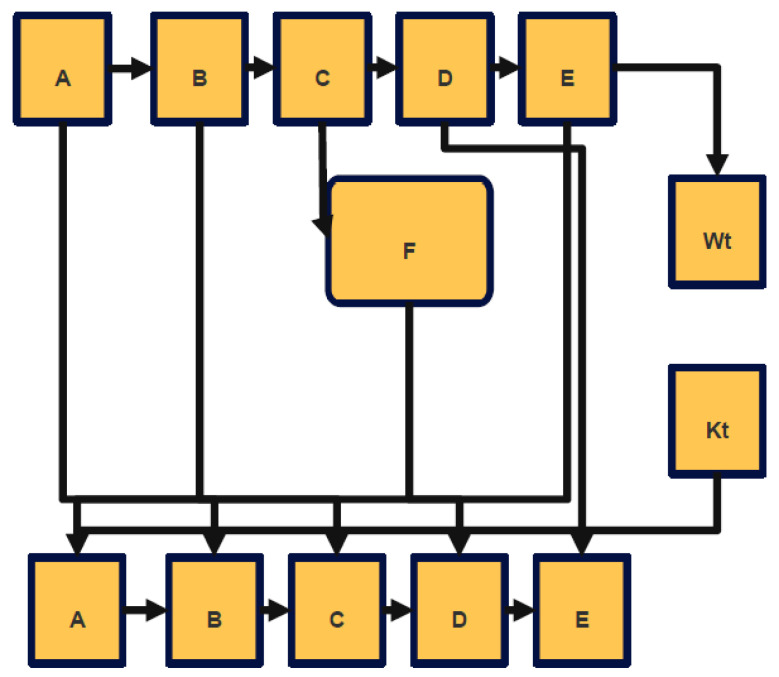
Iteration of SHA function.

Figure 6 represents the Iteration of SHA function of the proposed work.

##### SHA2 (SHA-256) Algorithm

In August of 2002, FIPS PUB 180-2 succeeded its predecessor, FIPS PUB 180-1, to become the new Secure Hash Standard. FIPS PUB 180-1 was previously in use. In 2005, security flaws in SHA-1 were found; more precisely, it was shown that a mathematical weakness may have existed, which implied that a more reliable hash function would be required. These findings led to the conclusion that SHA-1 needed to be replaced with another algorithm. Despite the fact that the SHA-2 algorithm shared some properties with the SHA-1 method, these attacks have not been successfully applied to the SHA-2 algorithm. The SHA-256 hashing method may process words that are 32 bits in length. Iterative one-way hash functions have the potential to process a message in order to produce a 256-bit message digest, which is a condensed version of the original message. This representation may be used to verify the legitimacy of the message. The technique makes it feasible to identify whether or not a message has been changed. If the message has been altered in any way, there is a very strong probability that the software will create a new message digest. This will occur whenever there is a change to the message. This quality is useful for creating random numbers, digital signatures, and message authentication codes, as well as verifying whether or not they were created successfully. In addition to that, it is helpful in the process of creating digital signatures (bits). The SHA-256 hashing method is used in the production of digital signatures.

##### Security of SHA-256

**Figure 7:**
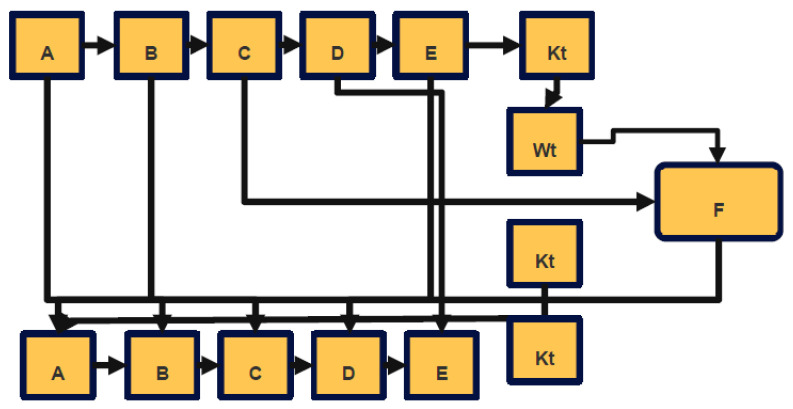
SHA compression function.

Only one of the two possible meet-in-the-middle preimage attacks that may be employed against SHA-2 makes use of the whole number of rounds. The first one carries out an attack on a SHA-256 that is 41 rounds long out of a total of 64 rounds, and the time complexity of this one is 2253.5, while the space complexity is 216. The second one launches an attack on a 42-round SHA-256, which has a time complexity of 2251.7 and a space complexity of 212. Figure 7 provides a look at one iteration of the SHA-256 compression technique; the whole algorithm may be seen here.

## 4. Results and Discussion

Each of these components is utilized in the method. The end result of processing a message using the SHA-256 algorithm is a digest that has 256 bits.

The designations W0 through W63 are used to indicate the individual words that are included in the message schedule. The letters A, B, C, D, E, F, and H are used to represent each of the eight working variables in this equation. SHA256 makes use of two different temporary words, and those words are T1 and T2.

This application makes use of a C implementation of SHA-256, and that implementation is mostly based on the following description, which is derived from the NSA’s original SHA-2 FIBS PUB 180-2. SHA-256 is a secure hashing algorithm.

Step 1: Adding Extra Fluff to the Message

The message, which will be represented by M, has to be padded before the computation of the hash value can begin. At this stage, it is absolutely necessary for the length of the padded message to be a multiple of 512 bits.

The next step is deciphering the padded message.

Before the hash calculation can begin, a message has to be parsed into N m-bit blocks after it has been padded. A total of N 512-bit words is extracted from the padded message.

(13) $$\tau_{ij}\left(t=0\right)=\frac{1}{\sum_{i=1}^{a}\;b_{i}}$$

Given that the 512 bits that make up the input block might be

(14) $$p_{ij\left(t\right)}=\frac{\tau_{ij\left(t\right)}\cdot \eta_{ij}}{\sum_{i^{\prime }}^{a}\;\sum_{j}^{bi}\;\tau_{ij}\left(t\right)\cdot \eta_{ij},\forall i\in I}$$

stated using sixteen words that are each 32 bits long, making up the initial 32 bits of the message block.

(15) $$\begin{array}{c} IG\left(t\right)=\;\sum_{i=1}^{m}\;P_{t}\left(C_{i}\right)logP_{t}\left(C_{i}\right)\;\;-\{P_{t}\left(t\right)\left[\sum_{i=1}^{m}\;P_{t}\left(C_{i}|t\right)logP_{r}\left(C_{i}|t\right)\right]\;+\\ P_{t}\left(t^{-}\right)\left[\sum_{i=1}^{m}\;P_{t}\left(C_{i}|t\right)logP_{t}\left(C_{i}|t^{-}\right)\right]\}\; \end{array}$$

$P_{z}\left(C_{i}|t^{-}\right)$: Conditional Complementary probability $P_{z}\left(C_{i}|t\right)$.

Comparisons of Various White Box Cryptographic Hash Functions is mentioned in Table 1.

## 5. Conclusions

As a consequence of the work that was performed in terms of research, innovative AES operations such as ECC-based S-box, SM-based mix column, and BEDT schemes for add round key were produced. These tactics were implemented with the support of white box cryptography systems after some time had passed. During the S-box creation process, which also includes ECC, BWMC is employed to discover flaws and apply remedies for those issues. Memory must maintain its reliability with a higher number of redundant bit operations, which is a drawback. This is a problem that must be solved. In further research, we may investigate other ways to reduce the number of unneeded bits while still ensuring that the memory remains consistent. It is likely that research on a better mix column technique that is based on a matrix may lead to improvements in the amount of processing time as well as the amount of physical space that is required. Within the framework of BEDT schemes for add round key, a comparison of a 2-bit transition to a 2-bit transition and an implementation analysis of the comparison are both carried out. Consideration is devoted to the transition from three bits to three bits; in the event that the idea is taken into account, additional attention may be given to BEDT systems as a potential option. The key size of the one-of-a-kind approach AES algorithm that was taken into consideration for the purpose of this study is 128 bits. However, the scope of the study could be broadened to include a variety of key sizes for the AES algorithm, such as 192 bits or 256 bits, as well as a performance analysis of the algorithm to determine the number of resources that are practically usable if it were implemented on white box cryptographic systems. White box cryptography offers a high level of security by making it difficult to decipher the secret key stored inside a cryptographic module. The cryptography module also has a few drawbacks, including poor performance and an issue with the key update for white box cryptography.

## Figures and Tables

**Table 1:** Comparison of various white box cryptographic hash functions.

			Internal State Size	Block Size	Length Size			Best Known Attacks (Complexity: Rounds)		
Algorithm	Year of Standard	Output Size (bits)				Word Size	Rounds	Collision	Second Preimage	Preimage
MD2	1989	64	384	128	-	32	864	Yes (2^63.3^)	Yes (2^73^)	Yes (2^73^)
MD4	1990	64	128	512	64	32	48	Yes (3)	Yes (2^64^)	Yes (2^78.4^)
MD5	1992	64	128	512	64	32	64	Yes (2^20.96^)	Yes (2^123.4^)	Yes (2^123.4^)
SHA-0	1993	64	160	512	64	32	80	Yes (2^33.6^)	No	No
SHA-1	1995	64	160	512	64	32	80	Yes (2^51^)	No	No
SHA-256 SHA-224	2002	126	256	512	64	32	64	Theoretical	Theoretical	Theoretical
	2004	126						l (2^28.5^:24)	l (2^248.4^:42)	l (2^248.4^:42)
SHA-512	2002	512	512	1024	128	64	80	Theoretical	Theoretical	Theoretical
SHA-384	2002	384						l (2^32.5^:24)	l (2^494.6^:42)	l (2^494.6^:42)

## Data Availability

The data supporting this study’s findings are available on request from the corresponding author.
